# Numerical simulation of geomechanical responses during horizontal-well cyclic thermal stimulation of natural gas hydrates

**DOI:** 10.1038/s41598-026-58898-w

**Published:** 2026-06-22

**Authors:** Panpan Zhang, Wenbin Gao, Zhenning Si, Zongjie Mu, Yawen Tan

**Affiliations:** 1 China University of Petroleum (Beijing) at Karamay, Karamay, 834000 China; 2 Xinjiang Key Laboratory of Intelligent Petroleum Exploration and Engineering, Karamay, 834000 China

**Keywords:** Natural gas hydrates, Thermal huff-and-puff production with horizontal wells, THMC coupling, Soaking-time optimization, Reservoir stimulation, Geomechanical response, Energy science and technology, Engineering, Solid Earth sciences

## Abstract

Thermal huff-and-puff production using horizontal wells offers a promising strategy to overcome the limitations of depressurization in low-permeability marine gas hydrate reservoirs, where slow pressure propagation and insufficient heat supply constrain gas recovery. However, its productivity and geomechanical response are governed by strongly coupled thermal–hydraulic–mechanical–chemical processes, the underlying mechanisms of which remain poorly constrained. Here, based on reservoir parameters from the Shenhu area of the South China Sea, we develop a three-dimensional, two-way coupled THMC model and validate it against data from the second field production test. The model is then used to systematically investigate the effects of soaking time and reservoir permeability on production performance and multiphysics responses. The results show that neglecting geomechanical feedback substantially overestimates reservoir flow capacity and gas production potential. An optimal soaking time exists at approximately 20 days: moderate soaking improves heat-use efficiency and promotes hydrate dissociation, whereas excessive soaking intensifies heat loss into surrounding formations and the overburden, thereby weakening the stimulation effect. Increasing reservoir permeability markedly enlarges the mobilized reservoir volume and enhances gas production, but also amplifies the magnitude and spatial extent of formation subsidence. Overall, the coordinated optimization of moderate soaking and reservoir stimulation is critical for balancing productivity enhancement with geomechanical safety. This study provides a theoretical basis for understanding THMC coupling mechanisms and optimizing operational parameters in thermal huff-and-puff production of marine gas hydrates.

## Introduction

Natural gas hydrates (NGHs) are ice-like crystalline compounds formed under low-temperature and high-pressure conditions, in which methane molecules are trapped within cage-like lattices of water molecules. They occur widely in deep-sea sediments and permafrost regions^1–5^. Owing to their vast global resource potential, high methane content, and substantially lower carbon-emission intensity than coal and oil, NGHs are widely regarded as an important unconventional clean-energy resource with both strategic value and environmental advantages^3–6^. In marine settings, the Shenhu area of the South China Sea has become one of China’s key frontiers for NGH development, owing to its high resource abundance, favorable burial conditions, and existing field production-test experience^7–12^. However, converting NGHs from a prospective resource into economically recoverable reserves requires overcoming several critical technical barriers, particularly inefficient production and reservoir instability.

The primary challenges in NGH production arise from the low permeability and strongly nonlinear multiphysics coupling of hydrate-bearing sediments (HBS). Existing production strategies mainly include depressurization, thermal stimulation, inhibitor injection and CO₂ replacement, among which depressurization has become the most widely adopted approach because of its relatively simple operation and low implementation cost^6–16^. For low-permeability marine clayey-silt reservoirs, however, depressurization alone is commonly constrained by slow pressure propagation, limited dissociation kinetics and local cooling induced by the endothermic decomposition of hydrates, making sustained and efficient gas production difficult to achieve^11–17^. Hydrate dissociation also weakens the load-bearing sediment skeleton and increases the effective stress, thereby triggering geomechanical risks such as reservoir compaction, seafloor subsidence, wellbore instability and sand production^18–23^. Although the second gas hydrate production test in the South China Sea demonstrated the engineering feasibility of depressurization using horizontal wells, the average daily gas production over 30 consecutive days remained far below the level required for commercial development^8,17,24^. This suggests that a single production method is insufficient for efficient marine NGH exploitation, and highlights the urgent need for enhanced recovery technologies that can simultaneously improve productivity and maintain reservoir stability.

In recent years, reservoir stimulation and thermal-enhancement strategies for improving NGH productivity have attracted increasing attention. Previous studies have shown that hydraulic fracturing, well-pattern optimization, multilateral-well development and boundary-condition regulation can enhance reservoir mobilization by improving near-wellbore flow conductivity, expanding the pressure-disturbance region and strengthening late-stage heat replenishment^25–33^. Thermal stimulation can provide external energy compensation for the endothermic dissociation of hydrates, and therefore holds substantial potential for low-permeability reservoirs^13,30–36^. As a production strategy that integrates heat supply with cyclic operational control, thermal huff-and-puff using horizontal wells is expected to improve the temperature–pressure distribution around the wellbore through repeated “heat injection–soaking–production” cycles, thereby promoting hydrate dissociation and optimizing gas–water production behavior. In addition, permeability enhancement induced by near-wellbore reservoir stimulation may further improve gas productivity by facilitating fluid migration and heat transfer^27–33^. Compared with depressurization-based production and fracturing-enhanced recovery^25–33^, studies on thermal huff-and-puff production of NGHs using horizontal wells remain relatively limited, particularly with respect to the coupled mechanisms between soaking-time optimization and reservoir stimulation.

Most existing numerical studies on thermally enhanced NGH production have primarily adopted thermal–hydraulic–chemical coupling frameworks, focusing on the evolution of temperature, pressure and phase-transition behavior, while insufficiently accounting for geomechanical responses and their feedback on fluid flow and heat transfer^18–21^. Although some studies have incorporated geomechanical analysis, they commonly rely on one-way coupling schemes, which cannot fully capture the reverse constraints imposed by pore-structure evolution, permeability reduction and increasing effective stress on long-term production behavior^18–23^. For low-permeability marine reservoirs, such treatment may lead to overestimation of production capacity and underestimation of risks associated with formation subsidence and wellbore instability^18–23^. Soaking time and reservoir permeability not only control heat-conduction depth and pressure-propagation range, respectively, but also jointly regulate the evolution of temperature, pressure, saturation and stress fields. It is therefore necessary to reassess their integrated effects on production performance and geomechanical response within a two-way coupled THMC framework^18–23^.

To address these issues, we develop a three-dimensional, two-way coupled THMC numerical model for thermal huff-and-puff production of NGHs using a horizontal well, based on representative reservoir parameters from the Shenhu area of the South China Sea. The numerical approach is validated against data from the second NGH production test in the South China Sea^8,37^. A series of simulation cases with different soaking times and degrees of permeability enhancement are designed to investigate gas and water production behavior, hydrate dissociation characteristics, pressure- and temperature-field evolution, and geomechanical responses such as formation subsidence during cyclic thermal stimulation. The differences between fully coupled THMC modelling and one-way coupling are further evaluated. The objective of this study is to reveal the controlling mechanisms by which soaking-time optimization and reservoir stimulation affect production performance in low-permeability marine NGH reservoirs, and to clarify the relationship between productivity enhancement and geomechanical response. The results provide a theoretical basis and parameter-optimization reference for the safe and efficient development of NGH reservoirs in the Shenhu area and similar low-permeability marine settings.

## Model construction

### Numerical method

**Fig. 1:**
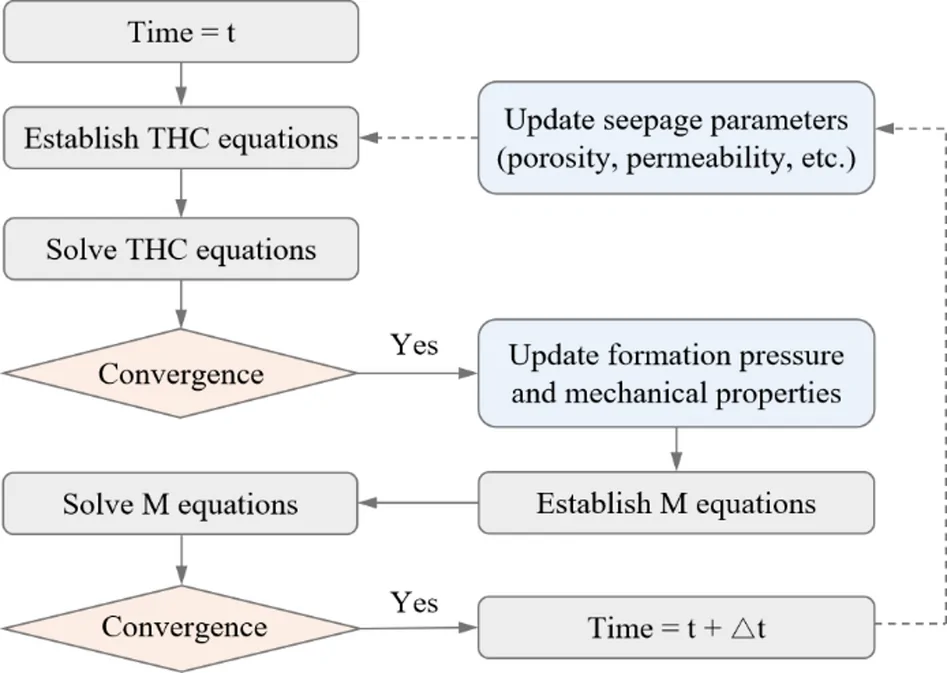
Schematic of the sequentially coupled THMC model with geomechanical feedback.

Gas hydrate production is a complex multiphysics coupling process. In this study, a THMC-coupled model for NGH production is established using the geomechanics module of CMG STARS^30^ to characterize the interactions among multiphase flow, heat transfer, hydrate dissociation and formation deformation. In the model, the thermal–hydraulic–chemical fields are fully coupled, while their coupling with the mechanical field is implemented in a two-way manner. Information exchange between the flow and mechanical fields is achieved through the GCOUPLING 2 option. Porosity is defined as a function of pressure, temperature and total mean stress, allowing deformation-induced pore-structure changes to be iteratively updated at the grid-block scale and fed back into the flow equations, thereby ensuring pore-volume consistency and mass conservation during time-step advancement^18–21^.Considering that near-wellbore regions and locally refined grids substantially increase the computational complexity of fully coupled solutions and reduce numerical convergence, the model adopts a solution strategy in which the THC fields and the mechanical field are two-way coupled within each time step. This approach is theoretically capable of producing results comparable to those of a fully coupled four-field THMC algorithm, while balancing computational accuracy and numerical stability^18–21^. The resulting three-dimensional, two-way coupled THMC numerical model can be used to simulate the co-evolution of temperature, pressure, phase-transition behavior and formation deformation during NGH production (Fig. 1).

### Model validation

The numerical model employed in this study is identical to that developed and validated in our previous work^37^, where a multiphysics coupled framework was established to simulate gas hydrate production in multilayered sediments of the South China Sea. As detailed in that study, the model was validated against field production data from the second offshore hydrate production test, demonstrating its capability to reproduce gas production behavior and the associated multiphysics responses.

Since the governing equations, numerical implementation, and reservoir parameterization remain unchanged in the present work, the previously validated model is directly adopted here. The focus of this study is therefore not on re-validation of the model itself, but on extending its application to cyclic thermal stimulation (CTS) processes, which introduce additional heat injection and shut-in mechanisms.

For completeness, a brief comparison with the field test data is retained. The simulation reproduces the characteristic production trend observed in the Shenhu trial, including the rapid decline in gas production rate during the early stage. After 30 days, the simulated average gas production rate is 97.6 m³/(d·m), which lies within the reported field range of 95.7–114.8 m³/(d·m)^8,37^.

**Fig. 2:**
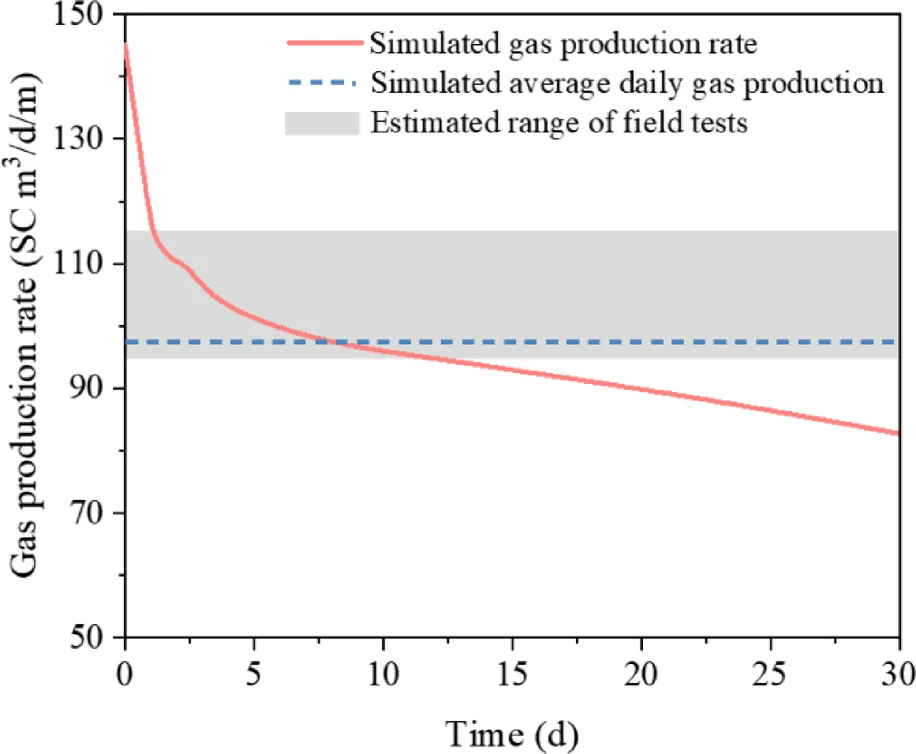
Comparison of simulated results with field production-test data^37^.

Where appropriate, the figure captions and labels have been revised to help identify the horizontal well location, pressure-disturbance region, heat-affected region, and maximum subsidence area more clearly (Fig. 2).

### Model description

**Fig. 3:**
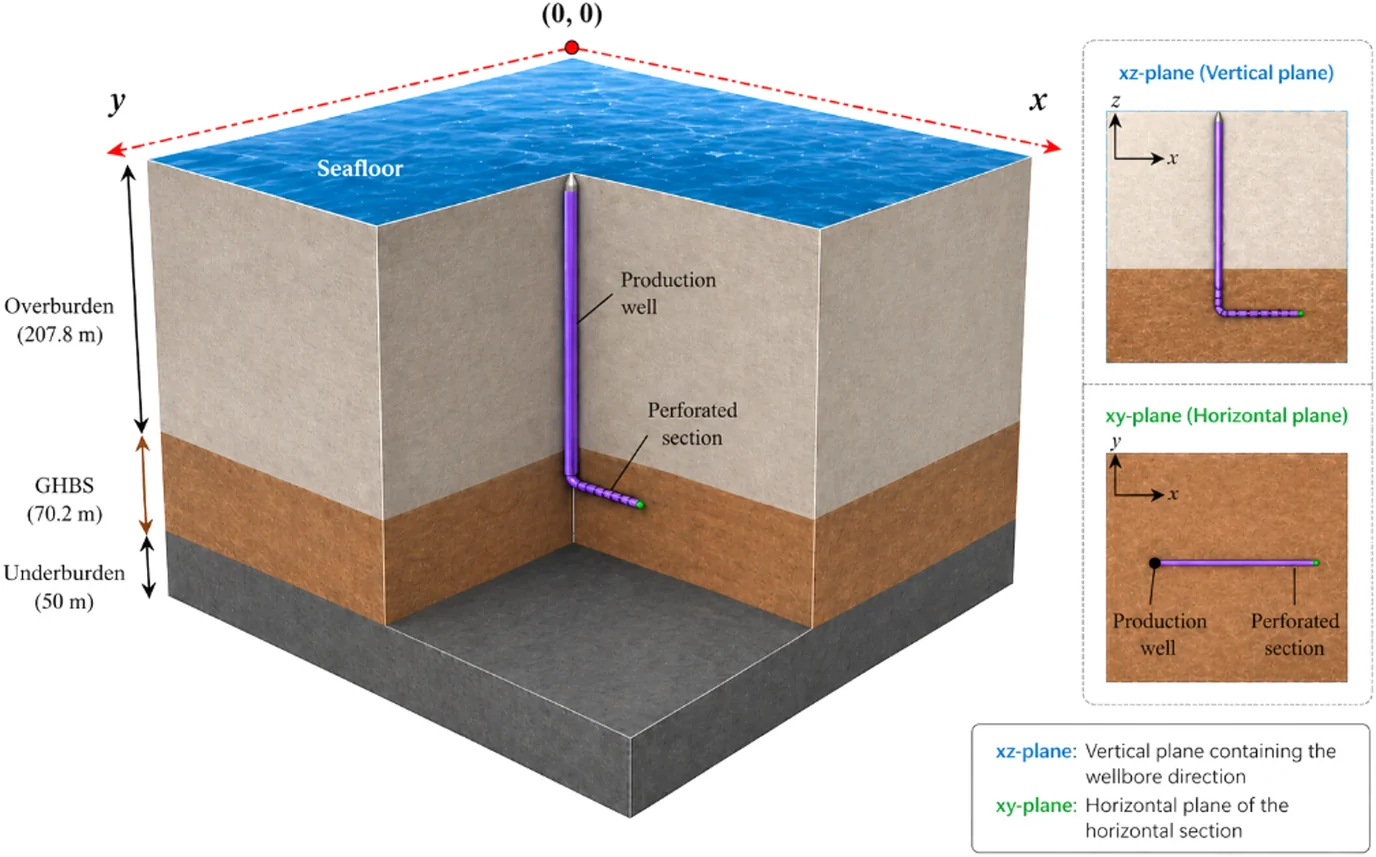
Schematic diagram of the two-way coupled THMC hydrate production model.

Figure 3 presents a schematic of the three-dimensional THMC model developed in this study^37^. The model was constructed by extending the previously validated two-dimensional framework, with several idealized assumptions introduced. Because the gas saturation in the free-gas layer is close to the immobile gas saturation and thus contributes only marginally to gas production, the free-gas layer was not included in the three-dimensional model. In addition, the initial temperature and pressure conditions of the three-phase layer, calculated from the geothermal gradient and seawater density, do not satisfy the three-phase equilibrium conditions for hydrate stability^1,2,38^. To ensure the stability of the model initial state, free gas in the three-phase layer was neglected, and this layer was simplified as an additional gas-hydrate-bearing sediment layer. This simplification may also influence the early pressure response and pressure-propagation characteristics due to the absence of pressure buffering and supplementary gas supply from free gas.

The three-dimensional model consists of a caprock layer and two gas-hydrate-bearing sediment layers, with the corresponding parameters listed in Table 1^7–10^. It should be noted that this study focuses on the effects of thermal huff-and-puff using a horizontal well on hydrate production. The contribution of initial free gas in the actual reservoir to gas production is therefore not considered in the model. Consequently, the predicted gas production may be lower than that expected under real engineering conditions.

**Table 1:** The layer settings of the 3D model.

Layer	Thickness (m)	Saturation	Porosity	Permeability (mD)
Overburden	207.8	S_w_: 1	0.37	2.38
GHBS	24.6	S_h_: 0.35 S_w_: 0.65	0.35	5
Underburden	50	S_w_: 1	0.35	6.80

The three-dimensional numerical model has dimensions of 450 m, 450 m and 328 m in the x-, y- and z-directions, respectively. It is discretized using a Cartesian grid comprising 68,850 grid blocks (45 × 45 × 34). To improve the resolution of flow and heat-transfer processes around the production well and fracture regions, local grid refinement is applied near the wellbore and fractures. The minimum grid size in the model is 2 m × 2 m × 1 m.

The top boundary of the model corresponds to the seafloor, with a water depth of 1,225 m^8^. The gas-hydrate-bearing sediment layer (GHBS) has a thickness of 70.2 m, while the overburden and underburden layers are 207.8 m and 50 m thick, respectively. A horizontal well is operated in a single-well injection–production mode and is placed in the middle of the GHBS, with a 100 m production interval. During production, the bottom-hole pressure is maintained at 1.5 MPa, and the injected steam temperature is set to 120 °C. A cyclic “heat injection–soaking–production” scheme is adopted to represent thermal huff-and-puff production conditions, with a total simulation period of three years^37^.

The initial conditions of the model are listed in Table 2, including key parameters such as porosity, permeability, hydrate saturation and thermal properties. The top and bottom boundaries are assigned constant-pressure conditions to represent external fluid supply from adjacent formations. Given the sufficient lateral extent of the model domain, the lateral boundaries are treated as no-flow boundaries for fluid-flow calculations.For the geomechanical model, normal displacement is constrained to zero at the lateral and bottom boundaries, while displacement in the other directions is allowed. Specifically, vertical displacement is fixed at the bottom boundary, and normal displacement is fixed at the lateral boundaries.

The constitutive behavior of hydrate-bearing sediments is jointly controlled by multiple factors, including hydrate saturation, sediment type, temperature and pressure. Accurately characterizing their mechanical response is therefore challenging, and constructing a fully representative constitutive model remains difficult^22,23^. Following previous studies, the mechanical properties of the gas-hydrate-bearing sediment layer (GHBS), such as cohesion and shear modulus, are simplified as linear functions of hydrate saturation. This treatment represents a common and widely adopted approximation in THMC-coupled simulations of natural gas hydrate systems^18–23^.

**Table 2:** Key parameters for simulating natural gas hydrate production^37^.

Parameter	Value	Parameter	Value
Effective stress gradient, kPa/m	8.30	Cohesion force (*S*_*h*_ = 1), MPa	2.0
Bulk modulus (*S*_*h*_ = 0), MPa	95	Hydrate-sensitive properties	$\:x={x}_{{S}_{h}=0}+c\cdot\:{S}_{h}$
Bulk modulus (*S*_*h*_ = 1), MPa	670	Friction angle, °	30
Shear modulus (*S*_*h*_ = 0), MPa	87	Dilatation angle, °	10
Shear modulus (*S*_*h*_ = 1), MPa	612	Biot coefficient	1
Cohesion force (*S*_*h*_ = 0), MPa	0.5	Yield criterion	Mohr-Coulomb
Hydrate type	CH_4_•5.75H_2_O	Hydrate dissociation	Kinetic model ^38^
Initial pressure, MPa (At the bottom of GHBS 2)	15.24	Pore pressure gradient, kPa/m	10.15
Initial temperature, ℃ (At the bottom of GHBS 2)	17.43	Geothermal gradient, K/m	0.059
Thermal conductivity of rock, J/(m·day·℃)	2.74 × 10^5^	Thermal conductivity of hydrate, J/(m·day·℃)	4.32 × 10^4^
Thermal conductivity of water, J/(m·day·℃)	5.35 × 10^4^	Specific heat of rock, J/(kg·℃)	1000
Phase equilibrium	${P_{eq}} = \exp (38.98 - \frac{{8533.8}}{{{T_{eq}}}})$	Absolute permeability variation model	$k = {k_0}{(\frac{{{\phi _f}}}{{{\phi _{f0}}}})^{3.5}}\cdot{\left[ {\frac{{\left( {1 - {\phi _{f0}}} \right)}}{{\left( {1 - {\phi _f}} \right)}}} \right]^2}$
Capillary pressure	$$\begin{gathered} {P_{{\mathrm{cap}}}} = {P_0}{\left[ {{{({S^*})}^{ - 1/\lambda }} - 1} \right]^{1 - \lambda }} \hfill \\ {S^*} = ({S_A} - {S_{irA}} + 0.001)/(1 - {S_{irA}}) \hfill \\ \lambda = 0.45;{\text{ }}{P_0} = {10^5}{\mathrm{Pa}} \hfill \\ \end{gathered}$$	Relative permeability	$\:{k}_{rA}={\left[\frac{{S}_{A}-{S}_{irA}}{1-{S}_{irA}}\right]}^{n}$ $\:{k}_{rG}={\left[\frac{{S}_{G}-{S}_{irG}}{1-{S}_{irG}}\right]}^{nG}$ $\:n={n}_{G}=3.572;\:$ $\:{S}_{\mathrm{irA}}=0.3;\:{S}_{irG}=0.05$

### Case design description

Table 3 summarizes the eight numerical simulation cases, each with a total simulation period of three years. Cases 1–5 are designed to evaluate the effect of soaking time on production performance. All five cases adopt a cyclic heat injection–soaking–production scheme, in which the heat-injection and production periods are fixed at 30 days and 50 days, respectively, while the soaking time is set to 0, 10, 20, 30 and 40 days. Cases 6–8 are designed to assess the effect of reservoir stimulation. In these cases, the operational schedule is fixed at 30 days of heat injection, 20 days of soaking and 50 days of production, while the reservoir permeability is increased to 3, 5 and 10 times its initial value, respectively. By comparing the simulation results across these cases, the effects of soaking-time optimization and permeability enhancement on NGH production performance and potential geomechanical responses can be systematically investigated.

**Table 3:** Simulation case design.

Case	Permeability of GHBS (mD)	Soaking time (d)	Heat injection time(d)	Production time(d)
1	K = 5	0	30	50
2		10	30	50
3		20	30	50
4		30	30	50
5		40	30	50
6	K = 15	20	30	50
7	K = 25		30	50
8	K = 50		30	50

## Results and discussion

### Effect of bidirectional THMC coupling

**Fig. 4:**
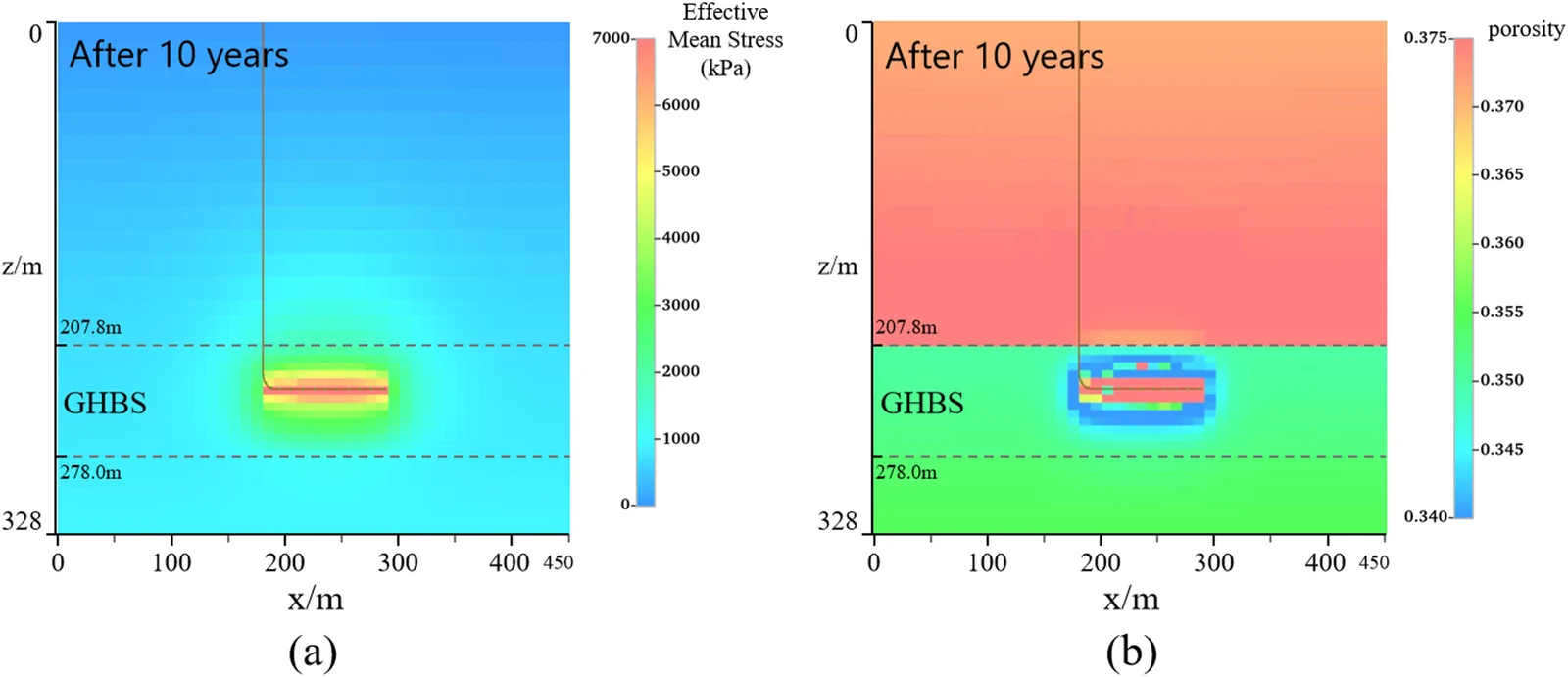
Diagram of geomechanical responses and porosity variation under two-way coupled THMC conditions.

**Fig. 5:**
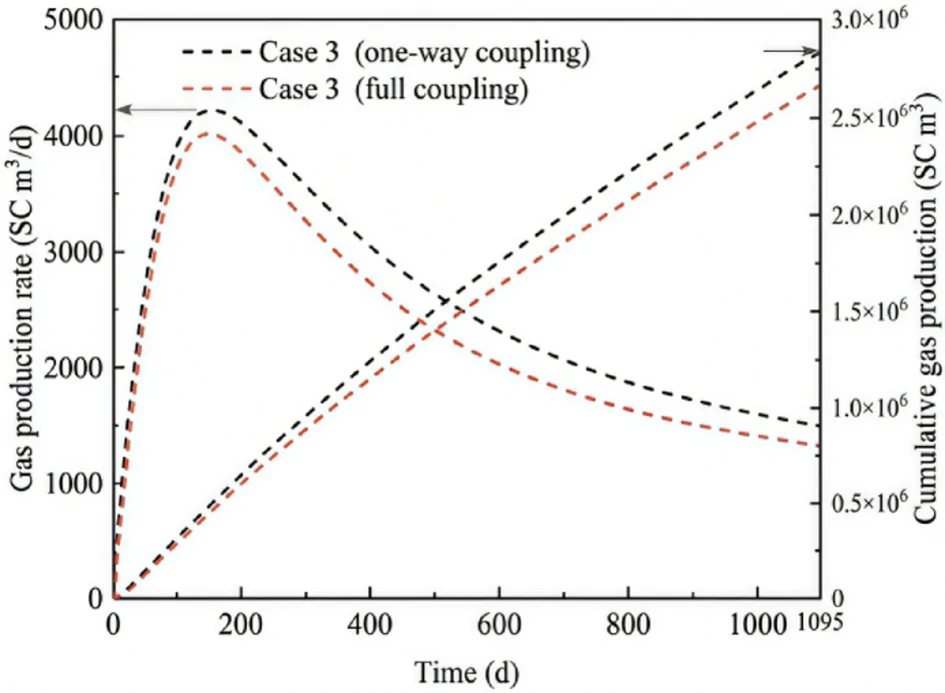
Effect of fully coupled on gas production behavior in hydrate exploitation.

Most studies on NGH production have adopted one-way coupling approaches, which do not fully account for the feedback of geomechanical responses on fluid-flow processes^18–21^. To quantitatively evaluate the effect of two-way coupled THMC model, the baseline thermal huff-and-puff case, Case 3, is used to compare the simulation results obtained from fully coupled and one-way coupled schemes. Figure 4 shows the spatial distributions of effective mean stress and porosity after three years of production under two-way coupled THMC model.The results indicate pronounced geomechanical responses in the formation surrounding the production well. Within the gas-hydrate-bearing sediment layer (GHBS), the effective mean stress increases markedly, with a local maximum of approximately 20,000 kPa, while porosity decreases. This indicates that depressurization and hydrate dissociation jointly induce significant formation compaction. The compaction effect is most pronounced near the wellbore and gradually weakens radially outward. As shown in Fig. 5, the peak gas production rate under fully coupled conditions is lower than that under one-way coupling, with values of approximately 4,100 and 4,250 SC m³/d, respectively. The cumulative gas production over three years also decreases under fully coupled conditions. This reduction is mainly attributed to geomechanical feedback associated with pore-structure evolution, which weakens reservoir flow capacity and consequently inhibits fluid migration. These results demonstrate that incorporating two-way coupled THMC model is essential for accurately predicting long-term production behavior and evaluating reservoir integrity during NGH exploitation^18–23^.

### Effect on gas production performance

**Fig. 6:**
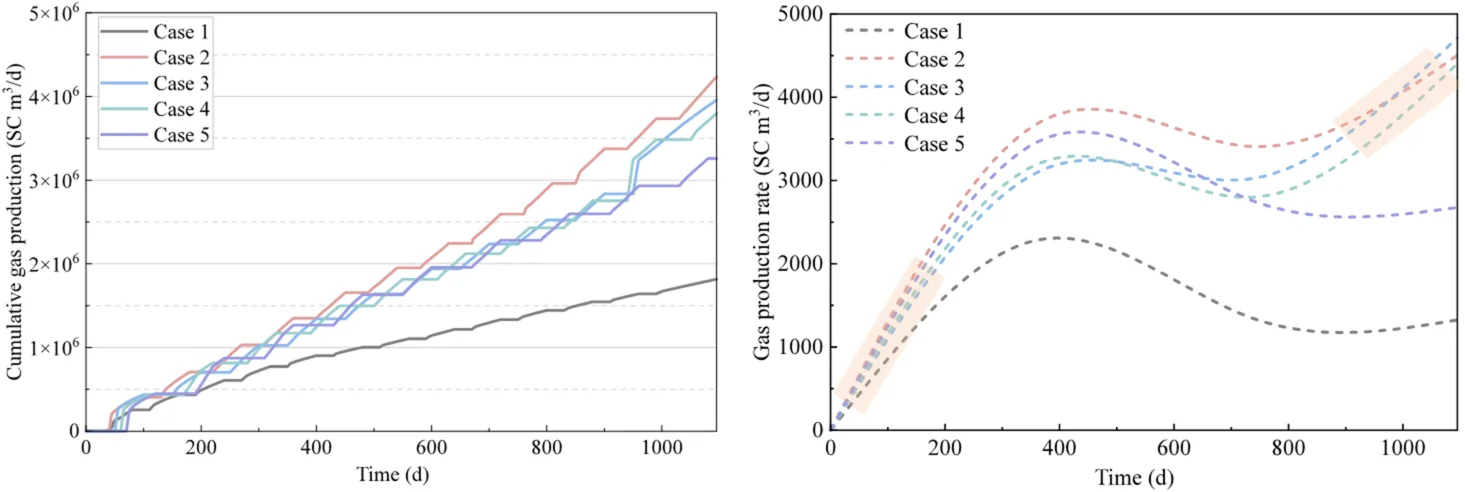
Temporal evolution of cumulative gas production and gas production rate in Cases 1–5.

**Fig. 7:**
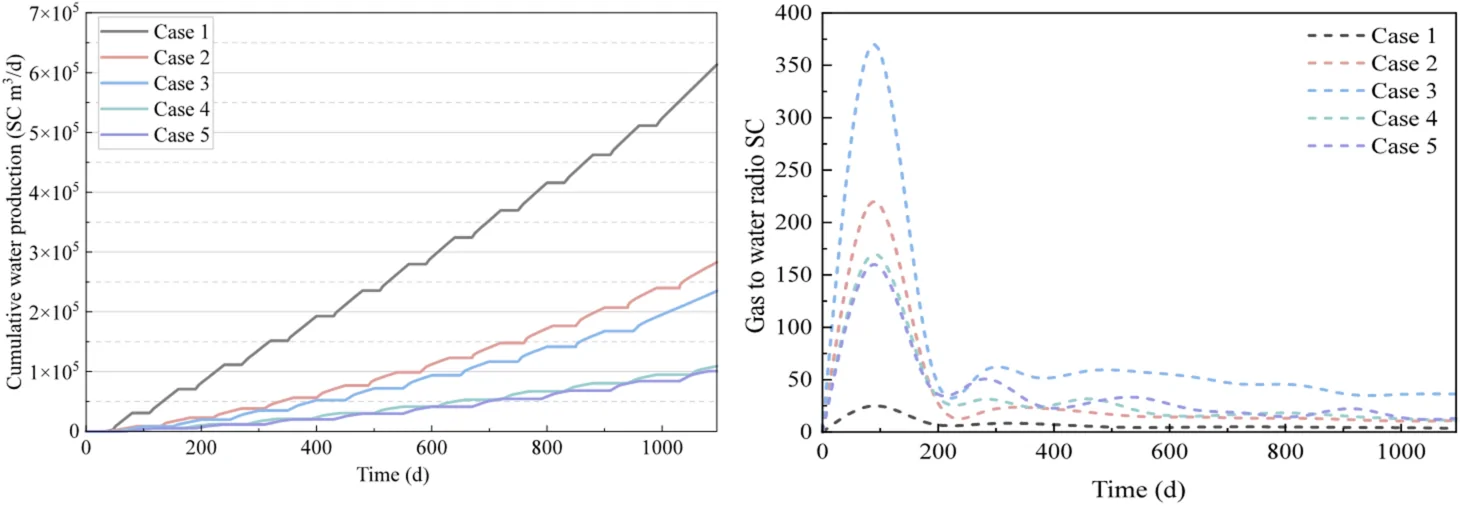
Temporal evolution of cumulative water production and gas–water ratio in Cases 1–5.

The temporal evolution of cumulative gas production and gas production rate differs markedly among the simulation cases, as shown in Fig. 6. At the end of the simulation, Case 1, without soaking, yields the lowest cumulative gas production and gas production rate. Case 2, with a soaking time of 10 days, achieves the highest cumulative gas production, whereas Case 3, with a soaking time of 20 days, shows a slightly lower cumulative gas production than Case 2 but the highest gas production rate. When the soaking time is extended to 40 days, as in Case 5, both cumulative gas production and gas production rate become lower than those in Cases 2–4. Figure 7 illustrates the temporal evolution of cumulative water production and gas–water ratio in Cases 1–5. These results indicate that a longer soaking time does not necessarily favor gas production. Beyond an optimal range, gas production declines, primarily because of changes in the transport and utilization of injected heat within the reservoir.

In Case 1, no soaking stage is included. Consequently, the heat injected into the reservoir is readily carried out by fluid convection during production and cannot effectively diffuse into deeper reservoir regions, resulting in a relatively low degree of hydrate mobilization within each cycle. In Case 3, the 20-day soaking stage allows the injected heat to diffuse into the surrounding reservoir during shut-in, effectively promoting thermodynamic re-equilibration around the wellbore and in the near-well region. This enhances hydrate dissociation and improves the accumulation efficiency of free gas, thereby yielding the best overall gas production performance. Although the extended soaking periods in Cases 4 and 5 further promote heat diffusion into the reservoir interior, their cumulative gas production does not increase and is instead slightly lower than that in Case 3. This indicates that the effect of soaking time has an optimal range: moderate soaking improves heat-use efficiency and promotes hydrate dissociation, whereas excessive soaking causes more heat to dissipate into the surrounding formations and overburden. When the additional heat loss exceeds the gas-production gain from further hydrate dissociation, the overall stimulation effect is weakened. Therefore, under the reservoir conditions and operational scenarios considered in this study, the 20-day soaking scheme exhibits comparatively favorable production performance.

As the number of huff-and-puff cycles increases, the gas production per cycle decreases in all cases, with the highest production occurring during the first cycle. This is mainly because readily dissociable hydrates in the near-wellbore region are preferentially produced during the initial stage. Subsequent cycles primarily act on reservoir zones farther from the wellbore and on residual hydrates, where heat-transfer efficiency and pressure response are relatively limited. As a result, production efficiency gradually declines with successive cycles. Overall, reservoir development exhibits a transition from rapid early-stage production enhancement to a later stage characterized by both production stabilization and decline.

Water production behavior also differs markedly among the cases. In Case 1, production starts immediately after heat injection, allowing the injected hot steam and liquid water generated by hydrate dissociation to be rapidly produced back. As a result, Case 1 shows the highest cumulative water production and the lowest gas–water ratio. In contrast, Cases 2–5 all include a soaking stage, which provides favorable conditions for further hydrate dissociation and free-gas accumulation, thereby improving gas–water production behavior and reducing ineffective water production. These results indicate that an appropriately designed soaking time not only enhances gas productivity, but also optimizes the gas–water balance during production, ultimately improving overall development efficiency.

**Fig. 8:**
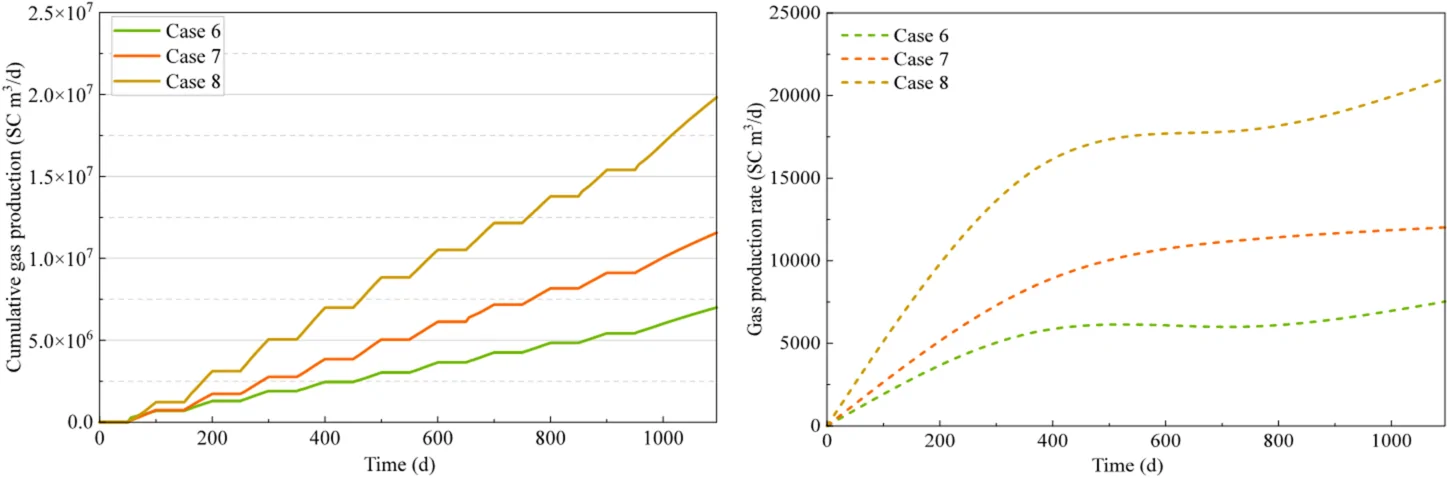
Temporal evolution of cumulative gas production and gas production rate in Cases 6–8.

Permeability enhancement exerts the most pronounced effect on gas production performance. As reservoir permeability increases in Cases 6–8, cumulative gas production rises substantially; by the end of the simulation, the cumulative gas production in Case 8 is approximately six times that in Case 1. This improvement occurs because increased permeability markedly expands the heat-affected region and pressure-propagation radius, enabling hydrates within a larger reservoir volume to be effectively mobilized and dissociated. In terms of production dynamics, as shown in Fig. 8, the high-permeability cases exhibit higher gas production rates and maintain relatively high production levels throughout each production cycle, indicating a substantial expansion of the mobilized reservoir volume. Although water production also increases considerably under high-permeability conditions, the concurrent improvement in gas productivity demonstrates that near-wellbore reservoir stimulation using horizontal wells can enable efficient development of low-permeability marine NGH reservoirs^25–33^.

### Effect on hydrate dissociation

**Fig. 9:**
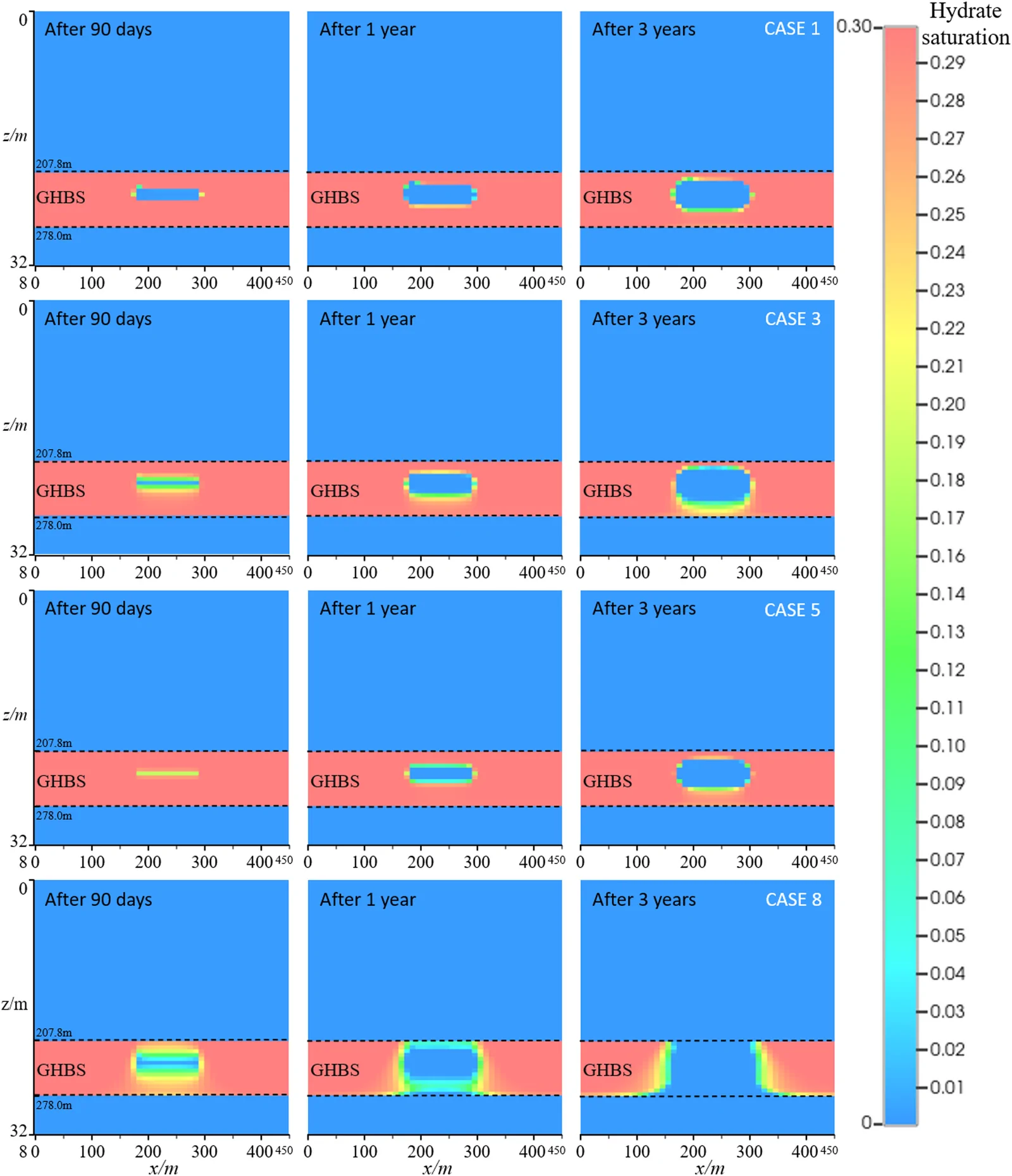
Evolution of hydrate saturation during production.

During thermal huff-and-puff production, hydrate saturation in the near-wellbore region of the horizontal well continuously decreases, while the dissociation extent, dissociation rate and residual hydrate saturation are jointly controlled by soaking time and reservoir permeability, as shown in Fig. 9. Extending the soaking time allows the injected heat to conduct more sufficiently through the reservoir, driving the hydrate dissociation front outward and reducing residual hydrate saturation around the wellbore. Compared with Case 1, Case 3, with a 20-day soaking stage, develops a larger low-saturation zone within the same production period. When the soaking time is further extended to 40 days, as in Case 5, the hydrate dissociation front continues to expand outward, accompanied by a further decrease in near-wellbore residual hydrate saturation. These results indicate that heat transfer during the soaking stage is dominated by conductive diffusion within the reservoir. The temperature gradient facilitates heat transfer to more distant regions, thereby promoting further hydrate dissociation. This enhancement is primarily reflected in the expansion of the dissociation region and the reduction of residual hydrate saturation; however, whether it translates into improved overall gas production over the full development period depends on additional controlling factors.

The enhanced reservoir permeability in Case 8 further increases the degree of hydrate dissociation. With improved fluid mobility, the migration range of thermal fluids and dissociated gas within the reservoir becomes broader, and heat and mass transfer become more efficient. Consequently, a low-saturation zone substantially larger than that in Case 3 is formed after the first cycle, accompanied by a more uniform dissociation pattern in the vertical direction. As production proceeds, the dissociation front in all cases continues to advance outward into the reservoir, but its propagation rate gradually decreases. This indicates that mobilizable hydrates in the near-wellbore region are preferentially consumed during the early production stage, whereas the ability of subsequent cycles to mobilize hydrates in far-wellbore regions becomes progressively weaker. By the end of the simulation, Case 1 retains the largest residual hydrate distribution, followed by Cases 3 and 5, with Case 5 showing a better overall dissociation performance than Case 3. In Case 8, hydrate saturation in most regions around the wellbore decreases to an extremely low level. Overall, both moderately extending the soaking time and increasing reservoir permeability help expand the hydrate dissociation front and improve the overall degree of reservoir mobilization. When these two effects act together, the enhancement becomes more pronounced.

### Effect on formation pressure and temperature

**Fig. 10:**
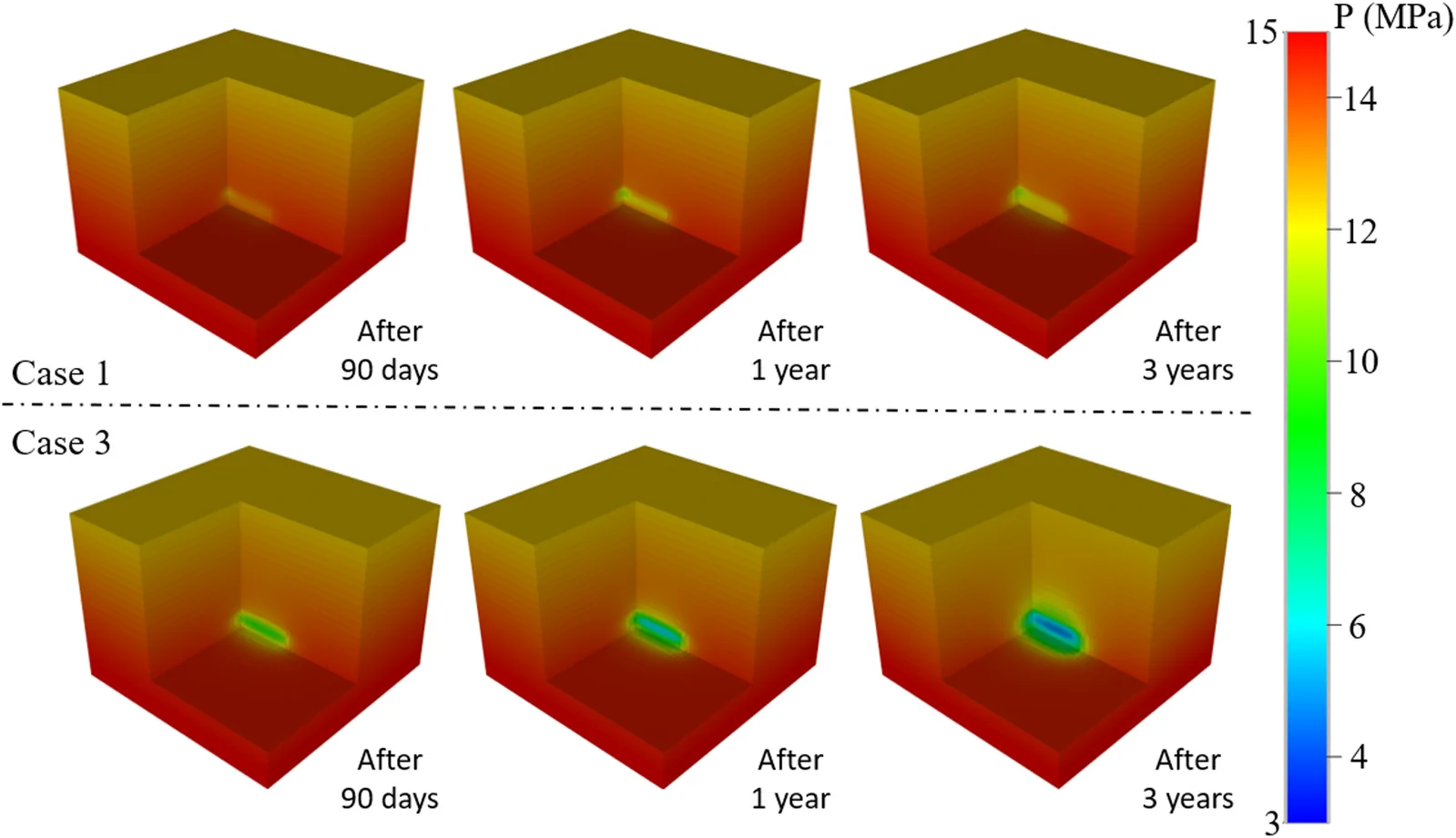
Three-dimensional cloud map of pressure dissipation and propagation in Cases 1 and 3.

**Fig. 11:**
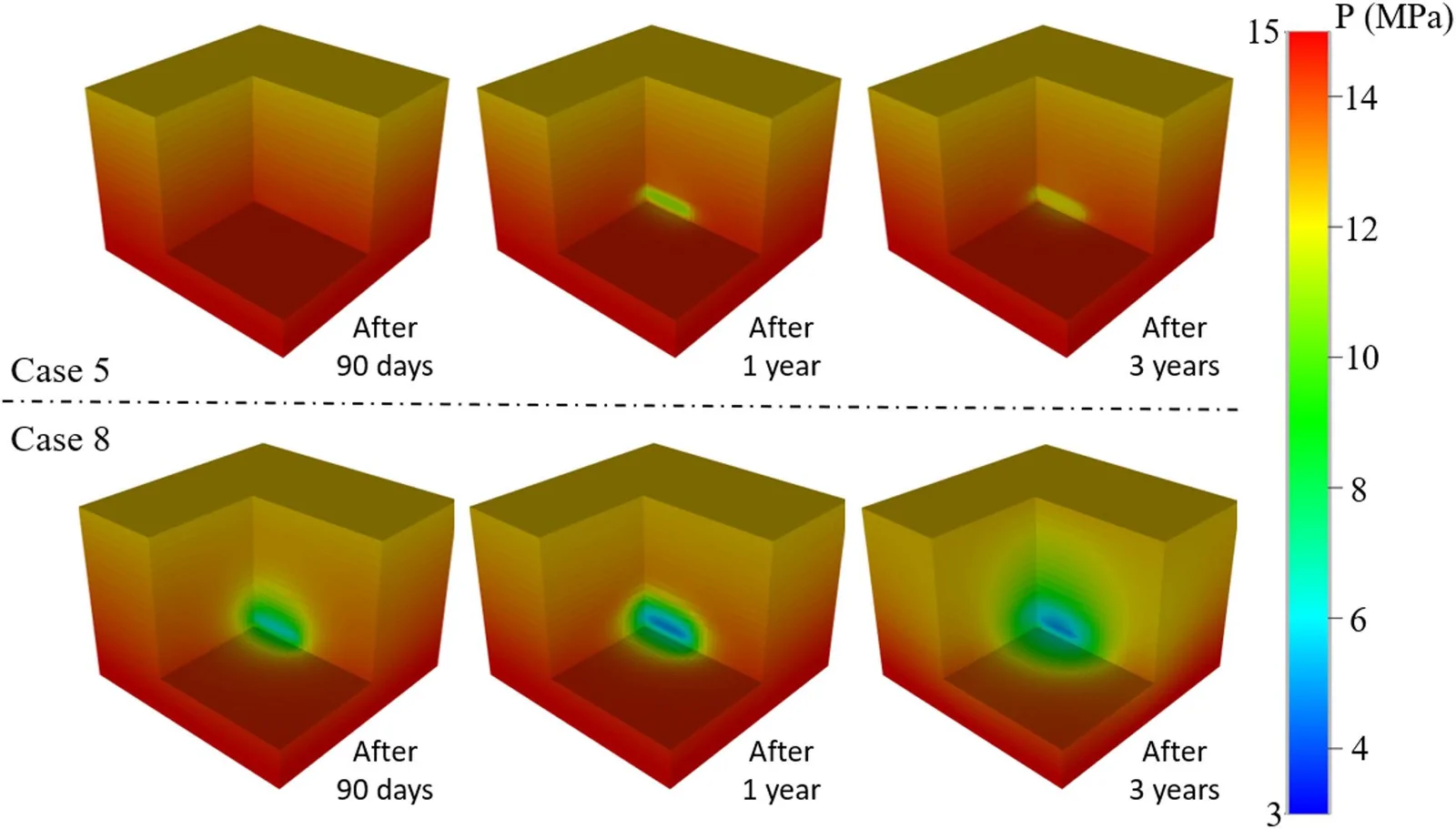
Three-dimensional cloud map of pressure dissipation and propagation in Cases 5 and 8.

**Fig. 12:**
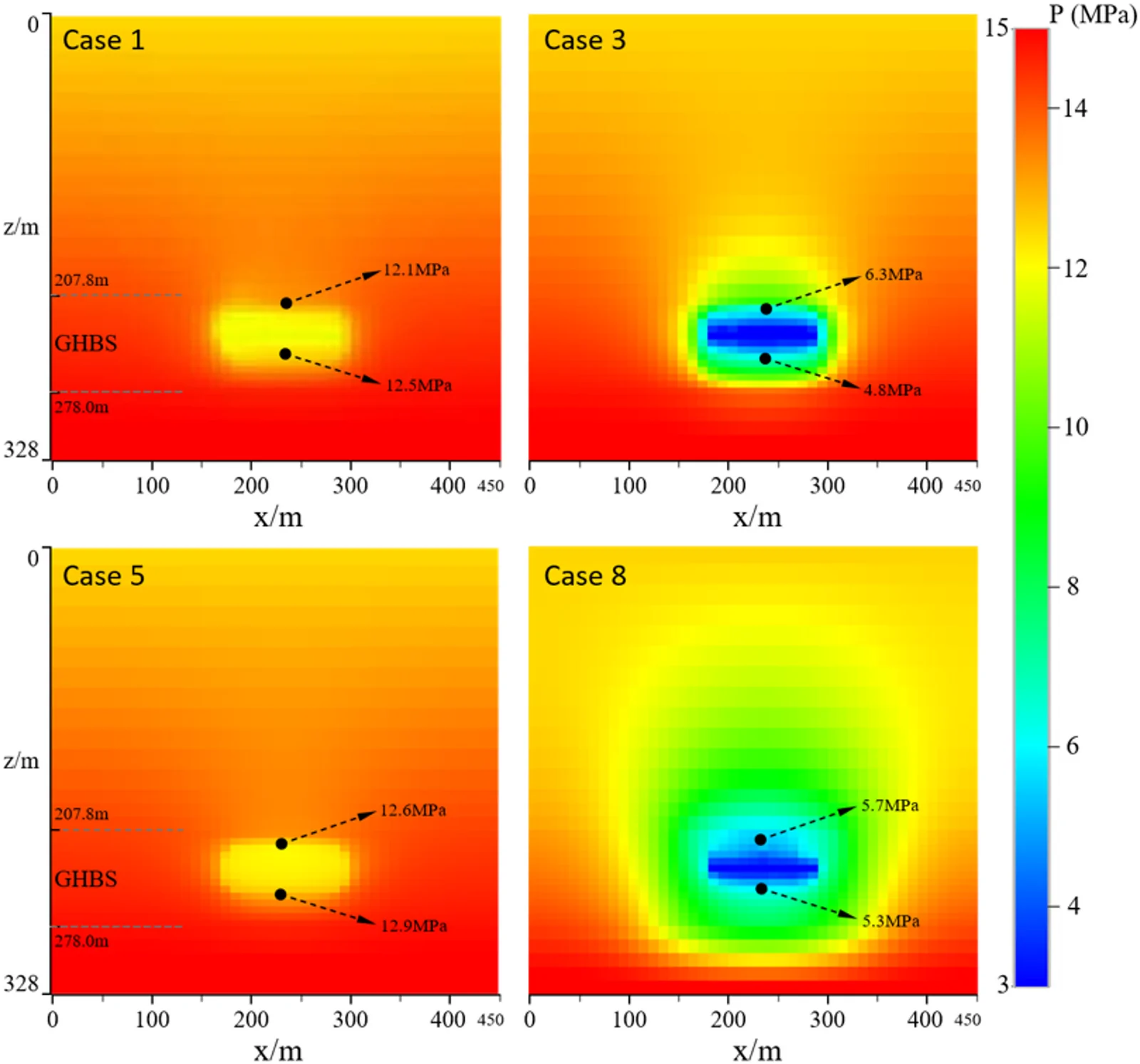
Vertical-plane pressure distribution after 3 years of production.

**Fig. 13:**
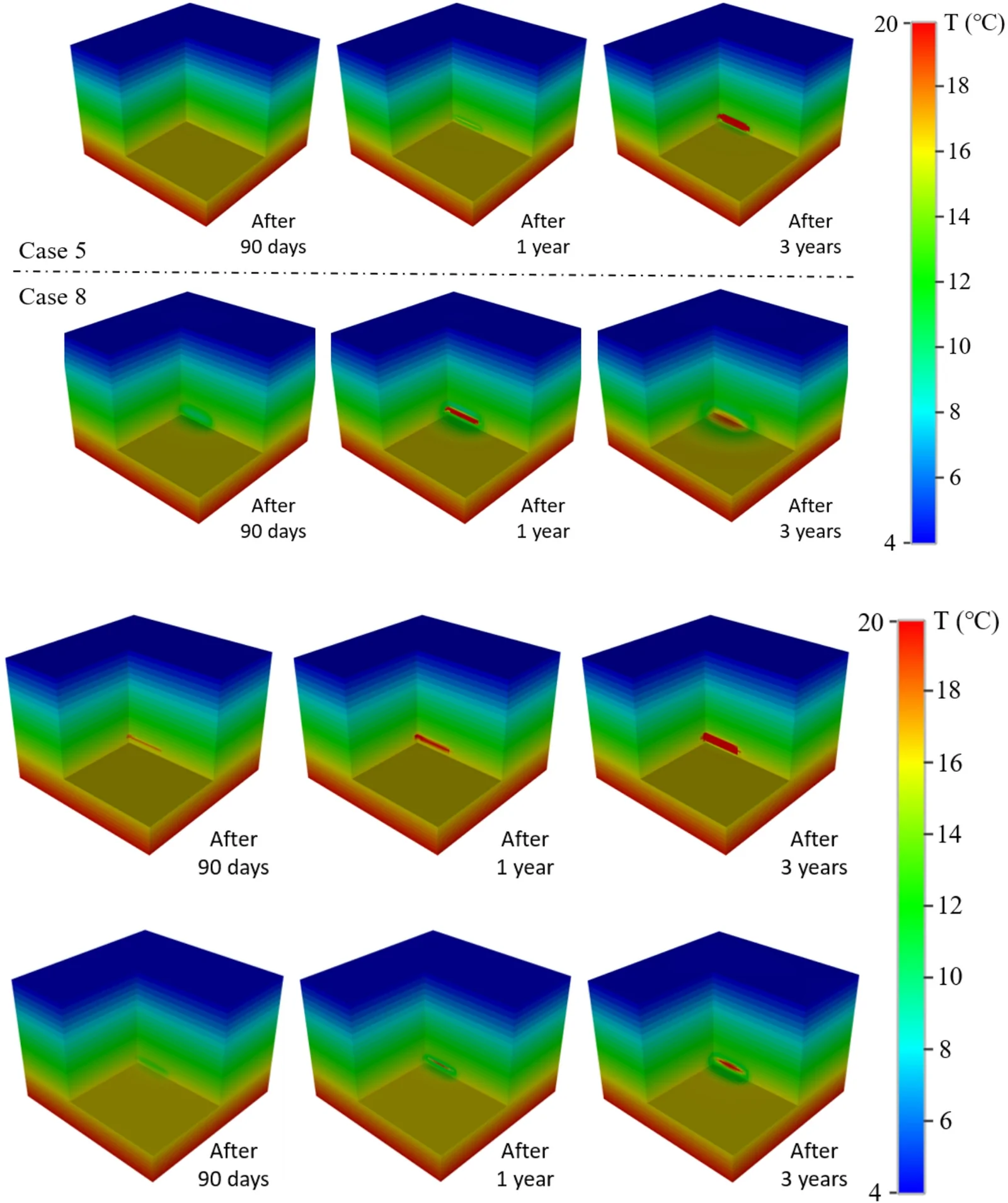
3D visualization of temperature diffusion and propagation.

**Fig. 14:**
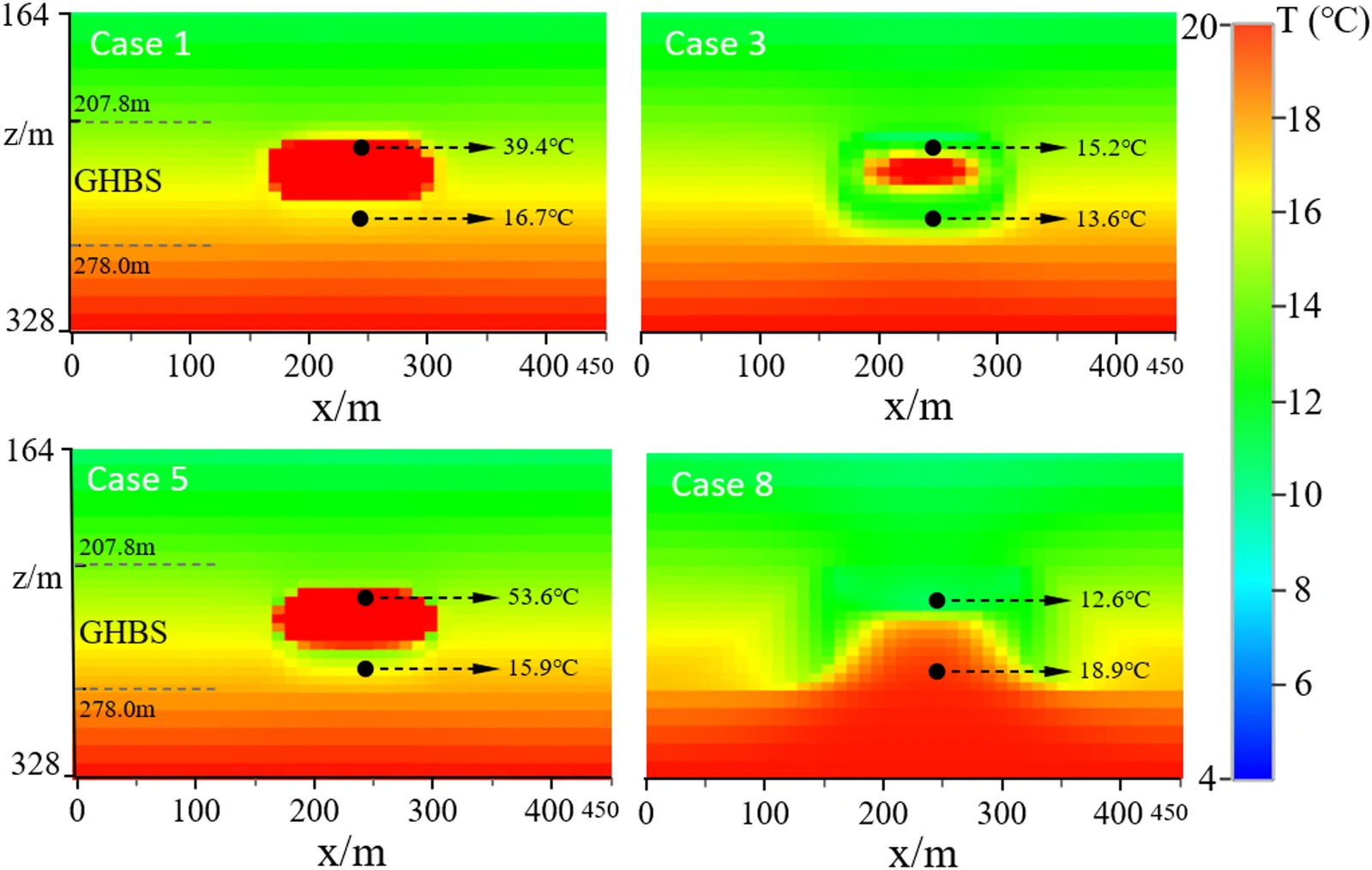
Vertical-plane temperature distribution after 3 years of production.

The evolution of the pressure and temperature fields around the horizontal well differs markedly among the simulation cases (Figs. 10, 11, 12, 13 and 14). During the first 30 days of heat injection, all cases rapidly develop a high-temperature, high-pressure zone near the wellbore. As production proceeds into the subsequent stages, however, the pressure- and temperature-field evolution diverges substantially under different operating schemes.In Cases 3 and 5, the well enters a soaking stage after heat injection and the wellbore is shut in. Under these conditions, the elevated pressure near the wellbore can be maintained for a certain period, and the pressure disturbance propagates outward primarily through slow radial diffusion. The formation of the pressure-depletion funnel is therefore delayed, and the overall pressure-gradient variation remains relatively moderate. By contrast, in Case 1, production begins immediately after heat injection. The bottom-hole pressure then declines rapidly, the near-well high-pressure zone dissipates quickly, and a low-pressure region develops earlier around the wellbore, resulting in a steeper pressure gradient.A comparison among the different soaking-time cases shows that Case 5, with the longest soaking time, maintains the elevated-pressure state for the longest duration and exhibits the largest spatial extent of pressure propagation, followed by Case 3, whereas Case 1 shows the smallest affected range. Under the high-permeability conditions of Case 8, local pressure variations are relatively more gradual, while both reservoir flow capacity and pressure-transmission capability are significantly enhanced, leading to the widest pressure-disturbance range.

The evolution of the temperature field is jointly controlled by heat-transfer mechanisms, the endothermic effect of hydrate dissociation and cooling induced by fluid expansion, as shown in Fig. 13. During the soaking stage, the injected heat in Cases 3 and 5 diffuses into the reservoir primarily by conduction, leading to a continuous expansion of the near-wellbore heat-affected region. Owing to its longer soaking time, Case 5 exhibits the largest heat-affected radius. In Case 1, production begins immediately after heat injection. High-temperature fluids are rapidly carried away from the near-wellbore region with the produced fluids, while low-temperature reservoir fluids flow toward the wellbore. As a result, both the temperature increase and the heat-affected region are smaller than those in the other cases. After the onset of production, the temperature field is further influenced by the coupled effects of endothermic hydrate dissociation and fluid-expansion cooling. In Case 1, depressurization occurs immediately at the early production stage, inducing relatively intense hydrate dissociation and making pronounced local cooling more likely. In Case 5, part of the hydrate has already dissociated during the soaking stage, which weakens the additional endothermic effect at the beginning of production and results in a relatively moderate cooling response. In Case 8, the high-permeability condition expands the heat-affected region, but also enhances the influx of low-temperature fluids toward the wellbore, leading to more pronounced near-wellbore cooling during long-term production^39^.

**Fig. 15:**
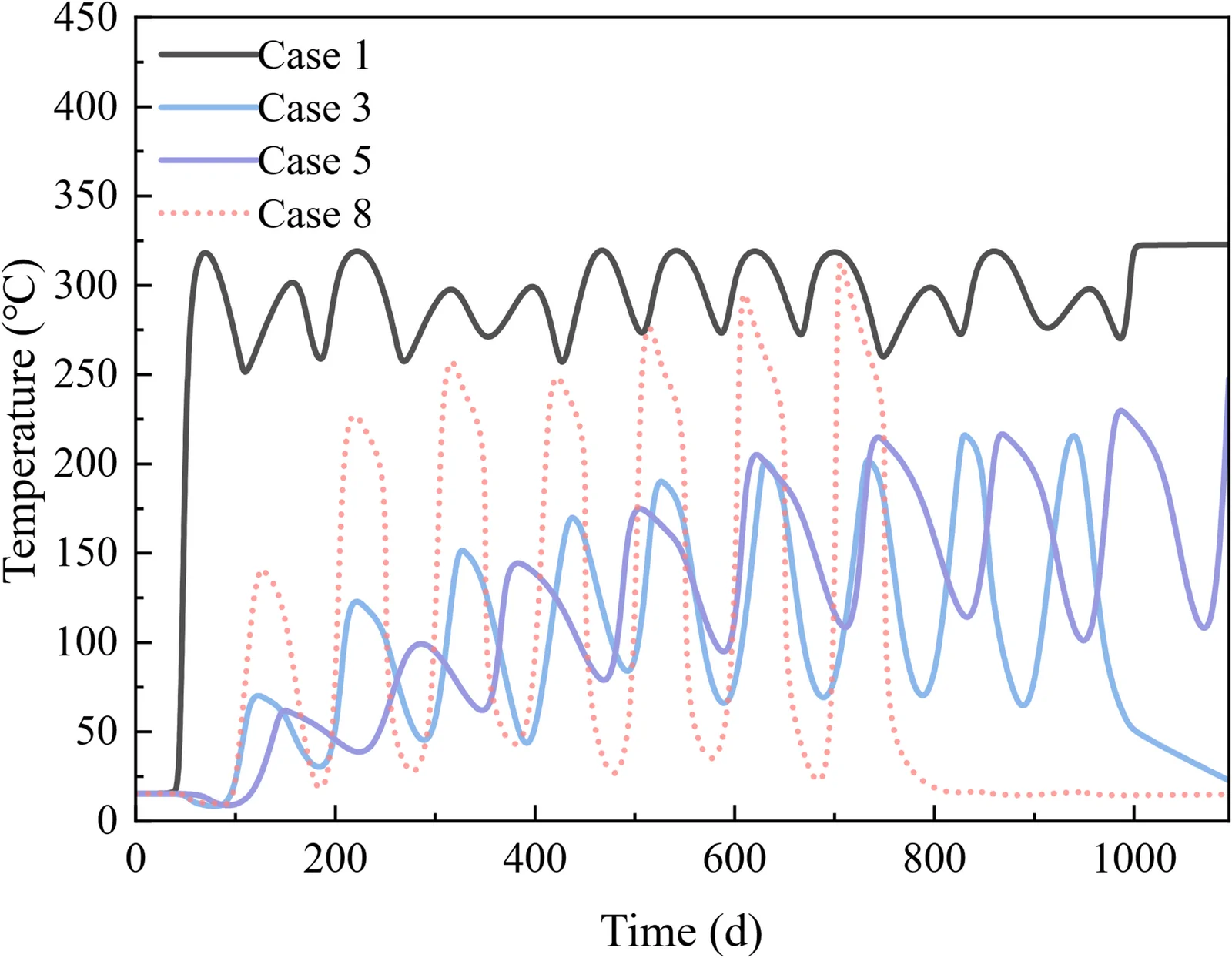
Temperature evolution at the monitoring point.

Figure 15 presents the temperature evolution at a monitoring point located in the middle section of the horizontal well for different simulation cases. The temperature curves in all cases exhibit pronounced cyclic fluctuations, corresponding to the cyclic operating scheme of thermal huff-and-puff production. During the heat-injection stage, temperature rises rapidly; during the soaking stage, temperature variation becomes more gradual as heat is conducted into the reservoir; and during the production stage, temperature decreases markedly owing to the combined effects of endothermic hydrate dissociation and low-temperature fluid influx. The differences among the cases are mainly reflected in the temperature peak, fluctuation amplitude and late-stage attenuation behavior.

In Case 1, no soaking stage is included. The injected heat is therefore rapidly carried away by fluid convection during production, resulting in a lower temperature peak and a relatively smaller overall fluctuation amplitude. By contrast, in Cases 3 and 5, the inclusion of a soaking stage allows the injected heat to be further conducted into the reservoir during shut-in, leading to a cycle-by-cycle increase in temperature at the monitoring point. Owing to its longer soaking time, Case 5 shows slightly higher temperature peaks and average temperatures than Case 3, indicating that moderately extending the soaking time helps improve heat-use efficiency.

The high-permeability case, Case 8, exhibits a faster thermal response, but also shows larger temperature fluctuations and more pronounced late-stage attenuation. This is mainly because increased permeability enhances fluid mobility and convective heat transfer, accelerating heat diffusion within the reservoir while intensifying the influx of low-temperature fluids toward the wellbore during production. The endothermic effect of hydrate dissociation further amplifies the temperature decline. Consequently, under high-permeability conditions, the temperature field is characterized by rapid heating and cooling, together with strong cyclic fluctuations.

The evolution of the temperature field reflects the combined effects of heat input, conductive diffusion, convective transport and endothermic hydrate dissociation. Moderate soaking improves the efficiency of heat utilization within the reservoir, whereas permeability enhancement, although capable of expanding the heat-affected region, may also accelerate heat dissipation and reduce the stability of the temperature field.

### Effect on formation subsidence

**Fig. 16:**
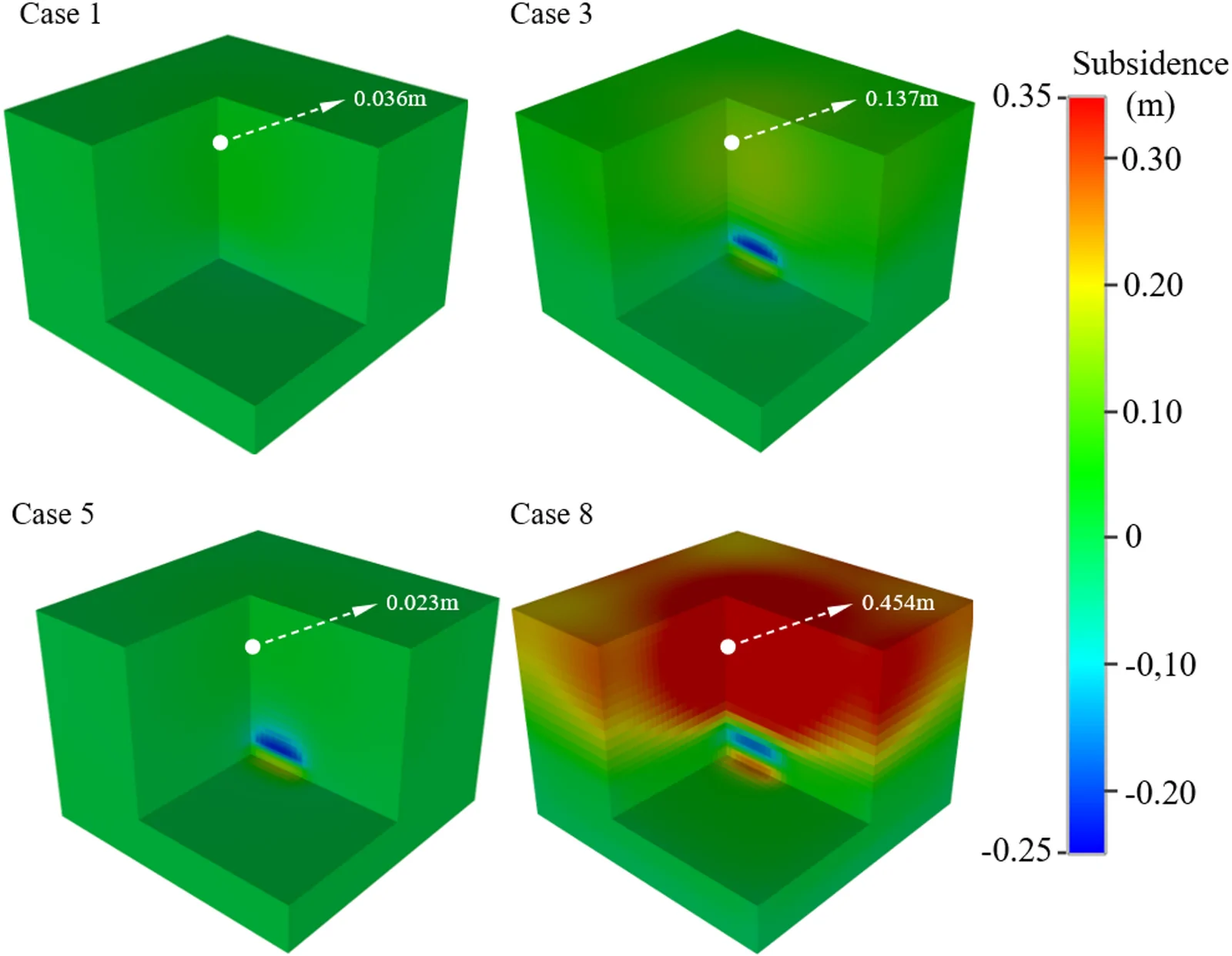
Schematic of formation subsidence after 3 years of production.

Hydrate dissociation and pore-pressure variations can induce geomechanical responses such as reservoir compaction, stress redistribution and formation subsidence, as shown in Fig. 16. Formation subsidence occurs in all cases, and the subsidence rate exhibits a “rapid-to-slow” temporal pattern. During the early production stage, the bottom-hole pressure decreases rapidly and substantial hydrate dissociation occurs, weakening the load-bearing capacity of the sediment skeleton and resulting in a relatively high subsidence rate. As the magnitude of pressure variation decreases, the subsidence rate gradually declines and the system enters a stage of slower deformation. The subsidence response differs markedly among the simulation cases. In Case 1, no soaking stage is included, and production starts immediately after heat injection. The near-wellbore pressure drawdown is therefore released more directly, leading to a pronounced stage-dependent subsidence pattern. In Cases 3 and 5, the soaking stage buffers the early pressure drawdown before production begins. Among them, Case 5 shows slightly greater subsidence magnitude and final subsidence than Case 3. Although Case 1 exhibits the most intense pressure-drawdown response, its final subsidence is slightly lower than that of Case 3 because of its lower cumulative gas production and weaker hydrate dissociation. The amplification effect of permeability enhancement on subsidence is more pronounced than that of soaking time. When reservoir permeability is increased by a factor of 10 in Case 8, the pore-pressure decline and effective-stress increase become more significant. The maximum subsidence above the wellbore reaches approximately two to three times that in Case 3, and the spatial extent of the subsidence basin expands markedly. This is mainly because, under high-permeability conditions, pore pressure dissipates more rapidly and the hydrate dissociation region becomes larger. Their combined effect further reduces the load-bearing capacity of the sediment skeleton, making the reservoir more susceptible to compaction deformation and substantially intensifying the overall subsidence response. These results indicate that although high permeability is beneficial for enhancing gas productivity, it can simultaneously aggravate geomechanical risks, including formation compaction, wellbore instability and sand production. Overall, permeability enhancement has a stronger influence on both production and deformation than soaking-time adjustment alone. Although the cumulative gas production in Case 8 is approximately six times that in Case 1, the maximum subsidence reaches approximately two to three times that in Case 3. This indicates that permeability enhancement substantially improves reservoir mobilization while simultaneously amplifying geomechanical risks, including formation compaction, wellbore instability and sand production.

In terms of spatial distribution, the subsidence troughs formed in all cases are located primarily above the horizontal well and exhibit an approximately elliptical shape. The maximum subsidence is concentrated near the well axis and gradually decreases with increasing radial distance. Cases 3 and 5 show similar subsidence patterns, whereas Case 1 has a smaller affected area, and Case 8 develops the deepest and widest subsidence basin. The subsidence response is not confined to the top boundary of the reservoir; evident vertical compression can also be observed near the reservoir base, indicating that formation deformation is not purely localized but shows a certain degree of interlayer transmission. From a geomechanical perspective, the phase transformation of hydrate from a solid phase to fluid phases weakens the overall mechanical strength of the reservoir, while pore-pressure reduction increases the effective stress, making local regions around the wellbore more susceptible to compaction and shear deformation. Case 8 exhibits the largest hydrate dissociation extent and the most pronounced reduction in reservoir strength, and therefore presents the highest risk of wellbore instability and sand production. By contrast, an appropriately designed soaking stage can buffer the transient pressure drawdown during the early production stage, mitigate abrupt strength degradation around the wellbore, and thereby improve wellbore stability.

From a geomechanical perspective, hydrate dissociation reduces the cementation and load-bearing capacity of hydrate-bearing sediments, while pore-pressure decline increases the effective stress. Their combined effect promotes compaction, stress redistribution, and potential local shear deformation around the horizontal well. Therefore, the simulated subsidence should not be regarded only as a deformation indicator, but also as an indicator related to wellbore stability, casing deformation, sand production, and potential seabed deformation. These risks become more pronounced under high-permeability conditions because the pressure-depletion region and hydrate-dissociation zone expand more rapidly.

### Discussion

Thermal huff-and-puff production using horizontal wells represents an important technological pathway for the development of marine natural gas hydrates. Its performance is jointly governed by the strong coupling among heat transfer, fluid migration and geomechanical responses^18–23,37,40^. Previous studies have mainly focused on the influence of individual factors on gas production behavior, whereas the synergistic effects of operational parameters, such as soaking time, and reservoir stimulation measures, such as permeability enhancement, remain insufficiently understood within a THMC-coupled framework^18–21^.In this study, a three-dimensional, two-way coupled THMC model is developed based on the reservoir conditions of the Shenhu area in the South China Sea. The model is used to investigate the mechanisms by which soaking-time optimization and reservoir stimulation influence gas production performance and geomechanical responses^18–23,37,40^. The findings provide new insights into the efficient development of low-permeability marine NGH reservoirs.

Soaking time plays a critical role in regulating development efficiency and heat utilization^13,30–36^. Moderate soaking promotes the conduction and diffusion of injected heat within the reservoir, allowing hydrate dissociation to progressively extend from the near-wellbore region to more distal reservoir zones. This process enhances gas production and increases the degree of reservoir mobilization. However, excessively long soaking increases heat dissipation into the surrounding formations and overburden, reducing effective heat-use efficiency and causing gas productivity to exhibit diminishing marginal returns.

Reservoir permeability enhancement exerts an even more pronounced influence on production performance. Increasing permeability improves fluid mobility and pressure-transmission efficiency, allowing pressure disturbances to propagate rapidly over a larger region while strengthening convective heat transfer. These effects expand the hydrate dissociation zone and increase both the gas production rate and cumulative gas production. In addition, permeability enhancement further amplifies THMC coupling effects by altering the evolution pathways of the pressure and temperature fields^18–21,27–33^.

From an engineering perspective, permeability enhancement should be constrained by both productivity improvement and geomechanical safety. Increased permeability expands the pressure-disturbance and heat-affected regions, thereby improving hydrate mobilization and gas production. However, it also accelerates pore-pressure dissipation, increases effective stress, weakens the hydrate-bearing sediment skeleton, and enlarges the subsidence basin. Therefore, reservoir stimulation should be optimized together with allowable subsidence, wellbore integrity, casing deformation risk, sand-production risk, and potential seabed deformation.

The hydrate production process is fundamentally controlled by the combined effects of heat conduction, convective heat transfer and endothermic phase transition^13,34–36,38^. During the soaking stage, heat conduction dominates, which is conducive to improving heat-use efficiency. Under high-permeability conditions, however, enhanced convective heat transfer accelerates heat propagation while also intensifying the influx of low-temperature fluids. This leads to stronger temperature fluctuations and reduced stability of the temperature field.With respect to the pressure field, permeability enhancement accelerates pore-pressure dissipation and expands the pressure-depletion region, thereby promoting depressurization-induced hydrate dissociation. However, it may also induce stronger spatially heterogeneous responses within the reservoir^18–21^.

Formation subsidence is primarily governed by the combined effects of pore-pressure dissipation and hydrate dissociation, and is strongly influenced by both the production scheme and reservoir properties^18–23^. The soaking stage can partially buffer the rapid pressure drawdown during early production, thereby mitigating the transient subsidence response. However, as the hydrate dissociation region continues to expand, long-term subsidence progressively increases. Permeability enhancement markedly intensifies pore-pressure dissipation and skeleton-strength degradation. Under the control of the Biot effective stress mechanism^41^, these effects induce stronger compaction deformation, leading to a substantial increase in both the magnitude and spatial extent of formation subsidence.

Under the low-permeability reservoir conditions of the Shenhu area, a coordinated development strategy that combines moderate soaking with reservoir stimulation is preferable. Moderate soaking improves heat-use efficiency, while controlled reservoir stimulation can expand the mobilized reservoir volume. However, excessive permeability enhancement may increase the risks of formation subsidence, wellbore instability and sand production.

It should be noted that this study has several limitations. A simplified constitutive relationship is adopted to describe the mechanical properties of the reservoir, and sediment heterogeneity and structural evolution are not yet fully considered^22,23^. In addition, sand production and wellbore-flow behavior are not further coupled in the model, which may lead to an underestimation of geomechanical risks during actual production^18–23^. The model also neglects the contribution of initial free gas and does not explicitly represent complex fracture-network structures^27–33^; therefore, the simulation results are conservative to some extent.Future studies should integrate field production-test data to further improve reservoir mechanical models and multiscale fracture-characterization methods. Moreover, the synergistic mechanisms among heat injection, reservoir stimulation and auxiliary heat-supply technologies should be explored to enhance the engineering applicability and predictive accuracy of numerical simulations.

The quantitative conclusions of this study are most directly applicable to Shenhu-type low-permeability marine hydrate reservoirs with similar clayey-silt lithology, hydrate saturation, permeability level, thermal properties, and stress conditions. The optimum soaking time, productivity improvement, and subsidence magnitude should not be directly generalized to hydrate systems with different lithology, permeability, stress regime, hydrate occurrence pattern, or well configuration without recalibration.

## Conclusion

In this study, a three-dimensional, two-way coupled THMC numerical model for thermal huff-and-puff production of natural gas hydrates using a horizontal well is developed based on reservoir parameters from the Shenhu area of the South China Sea. The model is used to investigate the mechanisms by which soaking time and reservoir permeability influence production performance and geomechanical responses. The main conclusions are as follows:

(1) Under the reservoir properties and operating schedule considered in this study, a soaking time of approximately 20 days provides a favorable balance between heat conduction, hydrate dissociation, free-gas accumulation, and geomechanical response. Moderate soaking improves heat-use efficiency and gas-water production behavior, whereas excessively long soaking increases heat loss into surrounding formations and weakens the marginal stimulation effect.

(2) Increasing permeability enhances fluid mobility and pressure-transmission efficiency, expands the pressure-disturbance range and heat-affected radius, and enables hydrates within a larger reservoir volume to be effectively mobilized and dissociated. Under high-permeability conditions, the peak gas production rate increases substantially, relatively high gas production levels are maintained throughout each production cycle, and cumulative gas production rises markedly.

(3) Temperature variation exhibits cyclic fluctuations consistent with the heat injection–soaking–production sequence, and its evolution is jointly controlled by multiple coupled mechanisms, including heat conduction, convective heat transfer, endothermic hydrate dissociation and fluid-expansion cooling. Soaking increases the diffusion depth and utilization efficiency of heat within the reservoir. Under high-permeability conditions, heat propagation is accelerated, but the influx of cold fluids and heat dissipation are also intensified, resulting in stronger temperature-field fluctuations.

(4) Permeability enhancement substantially accelerates pressure transmission, allowing pressure disturbances to propagate more rapidly and over a broader region within the reservoir. This promotes depressurization-induced hydrate dissociation and expands the dissociation front. By contrast, the soaking stage delays the pressure drawdown process, resulting in a more gradual evolution of the pressure field.

(5) Formation subsidence is jointly controlled by pore-pressure dissipation and hydrate dissociation, and is characterized by cyclic fluctuations superimposed on continuous accumulation. The soaking stage buffers the pressure-drawdown rate during early production and weakens the transient subsidence response. However, cumulative subsidence continues to increase as the hydrate dissociation region expands. Permeability enhancement markedly intensifies pore-pressure dissipation and the weakening of the reservoir skeleton, leading to a greater increase in effective stress and stronger compaction deformation. Consequently, both the magnitude and spatial extent of subsidence expand substantially under high-permeability conditions.

These conclusions are most applicable to Shenhu-type low-permeability marine hydrate reservoirs under the operating schedule considered in this study. Extension to reservoirs with different lithology, hydrate occurrence, permeability, stress regime, or well configuration requires recalibration of the model parameters and operating conditions.

## Data Availability

All data supporting the findings of this study are available within the paper and its Supplementary Information. The original references for all data used in this study are also listed therein.
